# Expression profiling of stemness markers in testicular germline stem cells from neonatal and adult Swiss albino mice during their transdifferentiation in vitro

**DOI:** 10.1186/s13287-024-03701-8

**Published:** 2024-04-01

**Authors:** Sivankutty Indu, Anandavally N. Devi, Mahitha Sahadevan, Jeeva Sengottaiyan, Asmita Basu, Shabith Raj K, Pradeep G. Kumar

**Affiliations:** 1 Rajiv Gandhi Centre for Biotechnology, Thycaud PO, Poojappura, Thiruvananthapuram, 695 014 Kerala India; 2 Department of Biotechnology, University of Kerala, Karyavattom Campus, Thiruvananthapuram, 695581 Kerala India

**Keywords:** Testis, Spermatogonia, Stem cell, Pluripotency, Differentiation, Embryoid body, *Oct3/4*, *Nanog*, *Nestin*, *Desmin*, *Alpha-fetoprotein*

## Abstract

**Background:**

Spermatogonial stem cells (SSCs) were considered to be stem cells with limited potencies due to their existence in adult organisms. However, the production of spermatogonial stem cell colonies with broader differentiation capabilities in primary germ cell cultures from mice of select genetic backgrounds (C57BL6/Tg14, ddY, FVB and 129/Ola) indicated that SSCs from these strains were pluripotent.

**Methods:**

We established primary cultures of SSCs from neonatal and adult Swiss 3T3 Albino mice. Stemness of SSC colonies were evaluated by performing real-time PCR and immunofluorescence analysis for a panel of chosen stemness markers. Differentiation potentials of SSCs were examined by attempting the generation of embryoid bodies and evaluating the expression of ectodermal, mesodermal and endodermal markers using immunofluorescence and real-time PCR analysis.

**Results:**

Spermatogonial stem cells from neonatal and mature mice testes colonised in vitro and formed compact spermatogonial stem cell colonies in culture. The presence of stem cell markers ALPL, ITGA6 and CD9 indicated stemness in these colonies. The differentiation potential of these SSC colonies was demonstrated by their transformation into embryoid bodies upon withdrawal of growth factors from the culture medium. SSC colonies and embryoid bodies formed were evaluated using immunofluorescence and real-time PCR analysis. Embryoid body like structures derived from both neonatal and adult mouse testis were quite similar in terms of the expression of germ layer markers.

**Conclusion:**

These results strongly suggest that SSC-derived EB-like structures could be used for further differentiation into cells of interest in cell-based therapeutics.

**Supplementary Information:**

The online version contains supplementary material available at 10.1186/s13287-024-03701-8.

## Introduction

Embryonic Stem Cells (ESCs) are pluripotent cells having the potential to differentiate into any type of cell in the body and can undergo differentiation in vitro by means of genetic modification or adding exogenous factors into the culture medium but may form teratomas in the accepting host [1]. Though ESCs offer wider potential for therapeutic applications in regenerative medicine, its use is hindered by the ethical concerns around the destruction of embryos for this purpose [2]. On the other hand, the use of adult stems cells in cell-based therapeutics is limited by their narrow range of potency, despite their potential for autologous stem cell donation, which may help to avoid issues of immune rejection [3]. Though pluripotency can be induced in somatic cells [4], both ESCs and iPSCs express several abnormalities during reprogramming and in prolonged culture. These include acquired abnormal karyotype [5–7], copy number variations (CNV) leading to mutations [8, 9], elimination of residual pluripotent cells [10] etc. iPSC induced teratoma was more aggressive than those induced by ES cells [11] and the molecular signature of iPSCs can be influenced by the cell type of origin [12]. iPSCs pose side effects related to transplantation in the accepting host, like formation of tumors due to the residual mass of pluripotent cells and hence their applications are limited currently. Recent research has established an optimized tool that will allow specific and selective removal of iPSCs by using LVCAGs iPSCs (transgenic) [1, 13].

Mammalian testis has a small population of Germ line stem cells (GSCs), which are descendants of primordial germ cells (PGCs) and have the ability to both self-renew and generate daughter cells that begin spermatogenesis [14]. Specification of PGCs consists of three main steps: repressing somatic programming, regaining the potential of pluripotency and epigenetic reprogramming in the entire genome. *Prdm14* is a transcriptional regulator which influences pluripotency and epigenetic reprogramming and is specifically expressed in the PSCs and germlines [15, 16]. GSCs constitute about 0.03% of the germ cells in the testis [17]. The genomic integrity of these cells appears to be higher because evolutionary selective forces act only on mutations of the germline genome and not on those in somatic cells [18]. Moreover, GSCs do not invoke the ethical concerns associated with the use of embryos for deriving embryonic stem cells (ESCs) for research [19]. Considering the advantages of GSCs over somatic stem cells, attempts have been made to derive pluripotent cells from GSCs. *In-vitro* proliferation of spermatogonial stem cells from testis of a new born transgenic mouse line C57BL6/Tg14 bred into DBA/2 background in growth factor supplemented media was reported in 2003 [20]. Two types of colonies showing typical characteristics of GS cell colonies and ES cell colonies were developed from neonatal ddY and DBA mice testis cultures [21]. SSCs from adult mice (C57BL/6, FVB and 129/Ola) testis responded to culture conditions and acquired embryonic stem cell properties and produced multipotent adult germline stem cells (maGSCs). Those maGSCs could generate three germ layers in vitro, produced teratomas in immunodeficient mice and could participate in development when injected into blastocysts [22]. Subsequently, putative SSCs and/or their progenitors were shown to reprogram to pluripotency when removed from their stem cell niche and when appropriate growth factors and reagents in embryonic stem cell medium are added [23]. In mice, PGC like cells (PGCLCs) were derived from ESCs and iPSCs by means of in-vitro culture through aggregates of Epiblast-like cells or EPiLc [16, 24]. Recent reports reveal the derivation of induced PGC-like cells (iPGCLCs) from mouse iPSCs, which could re-establish spermatogenesis following transplantation into the testis of infertile W/W^v^ mice [25]. As the derivation of SSC colonies depended on the genetic background [20], the differentiation capabilities of GSCs have been examined in selected mouse strains only. In this study, we have attempted to grow germline stem cells from testis of neonatal and adult Swiss albino mice and to evaluate their differentiation capability *in vitro.*

## Materials and methods

### Animals

Healthy male mice (*Mus musculus*, Swiss strain) bred in the institute animal facility, housed at temperature 27 ± 1° C and humidity-controlled conditions under 14 h light: 10 h dark and provided with food and water *ad libitum*, were used for the study. Neonatal (Postnatal day 12, P12), adolescent (postnatal day 30, P30) and adult (postnatal day 90, P90) male mice were used in this study. Use of laboratory animals for experiments was duly approved by the Institutional Animal Ethics Committee of Rajiv Gandhi Centre for Biotechnology, Thiruvananthapuram, vide Approval No. IAEC/66/PRK/2008.

### Reagents

Primary antibodies (GFRA1, ITGA6, ALPL, CD9, NES, TBXT and PDX-1), goat anti-rabbit IgG-FITC, goat anti-rabbit IgG- Alexa Fluor 488, Rabbit anti-goat IgG- Alexa Fluor 488, (Santa Cruz Biotechnology, CA, USA); CD9 antibody (BioLegend, San Diego, CA, USA); Reverse Transcriptase PCR primers (Sigma Genosys, Bangalore, India); Taq DNA polymerase, 100 bp & 1 kb ladder (New England Biolabs, MA, USA); DAPI, TRI reagent, Ethidium Bromide, agarose, glycine, Trizma Base and paraformaldehyde were purchased from Sigma- Aldrich, MO, USA.

### Preparation of single cell suspension from mouse testis

Neonatal and adult male mice were sacrificed by cervical dislocation and the testes were dissected out. Testis was then washed twice in sterile PBS and the tunica albuginea removed. A sequential enzymatic digestion was used to obtain single cell suspension of mice testis [26, 27]. The seminiferous tubules were digested with 1 mg/mL collagenase I and 5 µg/ mL DNase in Dulbecco’s Modified Eagle’s Medium (DMEM)/ F-12 supplemented with 1% Minimum Essential Medium, Non-Essential Amino Acids (MEM-NEAA) and 1% Antibiotic- Antimycotic solution for 20 min at 37° C. The tubes were kept at room temperature for 5 min to allow the seminiferous tubules to settle. The supernatant containing interstitial and peritubular cells was removed. The tubular fragments were subjected to a second digestion step with 1 mg/ mL collagenase I and 5 µg/ mL DNase and dispase solution (1mL/ 100 mg testis tissue) for 30 min at 32° C. The digestion mixture was gently agitated every 5 min during digestion to aid dissociation of the tubules. The digestion was stopped by adding DMEM /F12 FBS, mixed thoroughly and centrifuged at 270 × g for 5 min at room temperature. The supernatant was discarded and the pellet resuspended in DMEM/ F12 FBS. Filtration through a 40 μm cell strainer (BD Falcon, NJ, USA) removed the debri from the solution. The cells were observed under a microscope to ensure the absence of cell clumps and that the cells were intact.

### Primary cell culture of testicular cells

**Fig. 1:**
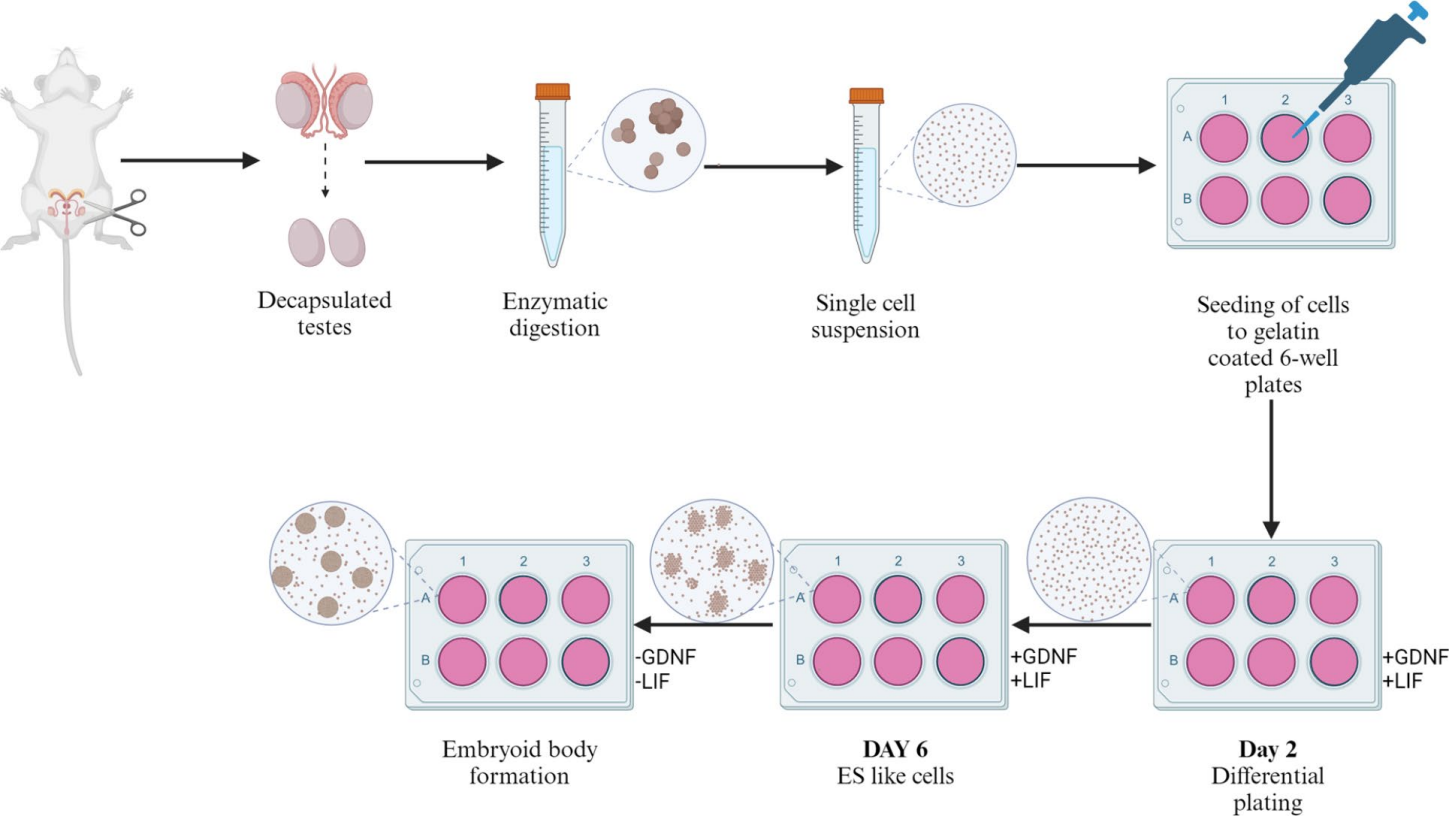
Graphical representation of the experiment design for generating ES-like colonies and embryoid like body development from spermatogonial stem cells harvested from the mouse testes (Created with BioRender.com)

Primary cultures of germ cells were established from neonatal and adult mice testicular cells [23, 26, 28]. The single cell suspension of testicular cells prepared as mentioned above was resuspended in SF medium supplemented with FBS (SF) [21, 22]. The SF medium composed of StemPro34 SFM base (Invitrogen, CA, USA) supplemented with StemPro34 nutrient supplement, non-growth factor components [5 mg/ mL Bovine serum albumin (Calbiochem, USA); 6 mg/ mL d-(+) Glucose, 10 µg/ mL d-Biotin, 25 µg/ mL Insulin, 30 µg/ mL Pyruvic acid sodium salt, 0.06% dl-Lactic acid (60% solution), 100 µM Ascorbic acid, 30 µM Sodium selenite, 60 µM Putrescine, 100 µg/ mL Bovine Apo-transferrin, 60 ng/ mL Progesterone, 30 ng/ mL β-Estradiol 17-cypionate, 10 µM 2-Mercaptoethanol (Sigma- Aldrich, MO, USA); 2 mM L-Glutamine, 1X Antibiotic–Antimycotic, 1X MEM vitamins, 1X Nonessential amino acids (Invitrogen, CA, USA); 1% Fetal bovine serum (Hyclone, Logan, USA)] and growth factor components [10^3^ U/ mL LIF, 10 ng/ mL Recombinant human basic FGF (Sigma Aldrich, MO, USA), 20 ng/ mL Recombinant mouse EGF and 15 ng/ mL Recombinant rat GDNF (R&D Systems, Minneapolis, MN)]. The resuspended cells were plated on a gelatin coated 12 well plate at a cell density of 2 × 10^5^ cells/mL and incubated for 16–24 h at 37° C in a humidified incubator with 5% CO_2_. The somatic cells of the testes attached to the gelatin plate and the germ cells did not. The floating germ cells were harvested using a P 1000 pipette and were transferred into a new 12 well plate after centrifugation at 270 × g for 5 min at RT. The germ cells were replenished with fresh SF medium every 3 days and the cultures were examined daily under a stereomicroscope for the appearance of SSC colonies The SSC colonies which started appearing by day 5 in culture were harvested using a micropipette and were placed in new 12 well plates containing fresh SF medium. Two such colonies were processed for immunofluorescence analysis of each of the stemness markers which was screened. The remaining colonies were transferred to new 12 well plates containing fresh SF medium [21] deprived of growth factors LIF and GDNF to induce embryoid body (EB) formation as reported earlier [29–31]. These cultures were maintained weeks in same medium for 2–3 [21] (Fig. 1).

### Immunofluorescence analysis of SSC colonies and embryoid bodies

SSC colonies and embryoid bodies were grown on poly-L-Lysine coated coverslips in a 12 well plate in appropriate medium at 37° C in a humidified incubator with 5% CO_2_ for 24 h. They were fixed with 4% paraformaldehyde in PBS, permeabilized in 0.1% Triton X-100 for 10 min and blocked for 30 min in 1% BSA. The SSC colonies were probed with antibodies against GFRA1, ITGA6, ALPL and CD9 (at a dilution of 1:200) for overnight at 4° C. Similarly, the embryoid body like structures were probed with antibodies against NES, TBXT (BRACHYURY) and PDX-1 (at a dilution of 1:200). They were further probed with fluorochrome conjugated goat anti rabbit (GFRA1, ITGA6, CD9) or rabbit anti-goat secondary (ALPL, NES, TBXT and PDX-1) antibodies (at a dilution of 1: 500) for 1 h. The dispersed cells from SSC colonies were counterstained with DAPI for 5 min at a final concentration of 0.50 µg/ mL to visualize the nuclei. The coverslips were washed in PBS, dried and mounted on glass slides using 60% glycerol. The cells were imaged on a Leica TCS-SP2 Confocal Microscope equipped with AOBS system (Leica, Mannheim, Germany).

### RNA preparation

Total RNA was prepared from SSC colonies/embryoid bodies derived from primary cultures of neonatal (one week old), adult (three months old) mice, using TRI reagent according to the manufacturer’s instructions. The SSC colonies/embryoid bodies were homogenized in 1 ml of TRI reagent (5 pulses at 1626 × g for 30 s each with 30 s intervals between pulses) using a PT-100 homogenizer probe (Kinematica AG, Luzernerstrasse, Lucerne). 200 µL of chloroform was added to the homogenate, incubated for 15 min and centrifuged at 12,000 × g for 15 min at 4° C. The upper transparent layer was transferred into a new eppendorf tube and RNA was precipitated using 0.5 mL isopropanol. The RNA pellet was washed in 70% ethyl alcohol, air dried and suspended in 35 µL sterile DEPC water.

### RT-PCR analysis

Single cell suspensions from the testes of P12, P30 and P90 mice were prepared as mentioned earlier. The cells were grown in DMEM/F12 medium supplemented with 10% FBS, 1% antibiotic–antimycotic and 1% non-essential amino acids (NEAA) in gelatin coated plates for 24 h. The floating germ cells were harvested on the following day using a P 1000 pipette, centrifuged at 270 × g for 5 min at RT and were transferred into a new low attachment 12 well plate. Further the cells were grown in DMEM/F12 medium supplemented with 10% FBS, 1% antibiotic–antimycotic and 1% NEAA without any growth factor. It was maintained and observed under microscope for 5–6 days. RNA was isolated from the SSC colonies derived from the testes from P12, P30 and P90 mice using Trizol reagent as explained above. 1 µg of RNA was converted to cDNA using Verso cDNA synthesis kit (Thermo scientific). Postnatal day 8 (P8) mouse testes cDNA was used as a positive control for this experiment. RT-PCR was performed for pluripotency factors such as *c-Myc, Klf4, Lin28, Nanog, Oct4* and Sox2 [4, 32–34]. The PCR products were resolved on 1.5% agarose gel. The expression level of beta-actin was used as an internal control. The primer pairs used for this experiment are listed in Table 1.

**Table 1:** Sequences of primers used in the real time and RT-PCR experiment

Serial No.	Gene Name	Primer Name	Primer Sequences (5’ – 3’)
1.	β-actin (*Actb*)	ba-560f	CTACCTCATGAAGATCCTGA
		ba-619r	TGATGTCACGCACGATTT
2.	Alkaline phosphatase2 (*Alpl*)	akp2-1089f	CGCCATGACATCCCAGAAA
		akp2-1168r	GGGTGTATCCACCGAATGTGA
3.	Glial cell line derived neurotrophic factor family receptor alpha 1 (*Gfra1*)	gfra-115f	GAACAGAGCTGCAGCACCAA
		gfra-194r	CCGGATGTCAGGCTGAAGTT
4.	Interferon induced transmembrane protein 3 (*Ifitm3*)	ifitm3-260f	GGAAGATGGTGGGTGATGTGA
		ifitm3-339r	GAGGACCAAGGTGCTGATGTTC
5.	Integrin α6 (*Itga6*)	intα6-977f	ATGCAGATGGGTGGCAAGAC
		intα6-1056r	GTAAACTGCACCCCCGACTTC
6.	*Nanog*	qnanog-771f	GCCTTACGTACAGTTGCAGCAA
		qnanog-850r	GCGCATGGCTTTCCCTAGT
7.	POU domain, class 5, transcription factor 1(*Pou5f1*)/*Oct3/4*	pou5f1-660f	GCAGGCCCGGAAGAGAAA
		pou5f1-739r	TCGGGCACTTCAGAAACATG
8.	DEAD (Asp-Glu-Ala-Asp) box polypeptide 4(*Ddx4*)	vasa-1787f	CCAGCTTCAGTAGCAGCACAAG
		vasa-1866r	GTGTGCTTTGCCCTGGTAATTC
9.	Dynein light chain 1(*Dynlt1*)	tctex-10f	TTCCAGGCCTCAGAGGAGACT
		tctex-89r	CCGATGGCGCTTTCTATAGC
10.	Desmin (*Des*)	desmin-75f	ATGAGCCAGGCCTACTCGTC
		desmin-167r	CTCGAGGGAACACGGGAGAG
11.	Alpha –fetoprotein (*Afp*)	AFP-1453 F	GAAGCAAGCCCTGTGAACTC
		AFP-1554R	GGCATAGGTTTCATCCCTCA
12.	Nestin (*Nes*)	nestin-4986 F	GGAAGAGAGTGGGGAAGAGG
		nestin-5101r	CATCCTGGACCTTGACACCT
13	*cMyc*	c-Myc F	CCTGTACCTCGTCCGATTCC
		c-Myc R	TTGTGTGTCCGCCTCTTGTC
14	*Klf4*	Klf4 F	GCCCAACTACCCTCCTTTCC
		Klf4 R	CCATGATTGTAGCGCTTGCC
15	*Lin28*	Lin28 F	GGGCTAGACCATCATGCCAA
		Lin28 R	ACTTGTTTCGCTTCCCGTCT
16	*Nanog*	Nanog F	AAATCCCTTCCCTCGCCATC
		Nanog R	ACCGCTTGCACTTCATCCTT
17	*Oct4*	Oct4 F	TGGCTTCAGACTTCGCCTTC
		Oct4 R	GAAGCGACAGATGGTGGTCT
18	*Sox2*	Sox2 F	ATGCACAACTCGGAGATCAG
		Sox2 R	GTTCATGTAGGTCTGCGAGC

The numbers mentioned in the names of primers indicate the start of the annealing region of each of the primers on the target sequence. The letters F and R represent the forward and reverse orientations of the primers. All the primers are written in the 5’- 3’ direction

### Real-time PCR analysis

Real-time PCR analyses were done for floating germ cell population (FGCP), spermatogonial stem cell clusters (SSCs) and SSCs derived embryoid bodies (EB) from the immature and adult testis. 1 µg RNA was reverse transcribed using SuperScript™ VILO™ cDNA synthesis kit (Invitrogen, Carlsbad, CA, USA) following manufacturer’s instructions. Primers used in the real time PCR experiment are listed in Table 1. PCR for pluripotency and germ cell markers and differentiation markers was done using a gene specific forward and reverse primer of each in a total of 5 µl reaction with SYBR green master mix (Applied biosystems, WA, UK) in ABI 7900 HT Sequence Detection System (Applied Biosystems, Netherlands) under standard qPCR temperature conditions. The following cycling parameters were used: 50º C for 10 min, 90^ο^ C for 10 min and 95^ο^ C for 10 min. This was followed by 40 cycles at 95^ο^ C for 10 s and a combined annealing/extension temperature of 60º C for 2 min. Expression level of beta actin was used as internal control. Two biological replicates, each with three technical replicates and with appropriate nontemplate controls (NTCs) were analysed. Fold changes were calculated using the comparative delta delta Ct (ΔΔCt) method for relative quantitation. Statistical analysis of these genes between the floating germ cell population versus SSCs, and SSCs of mature and immature testes versus SSCs derived embryoid bodies was done using two-tailed Student’s t-test on averaged Δ∆Ct values of these genes.

## Results

### Primary culture of mouse testicular cells

**Fig. 2:**
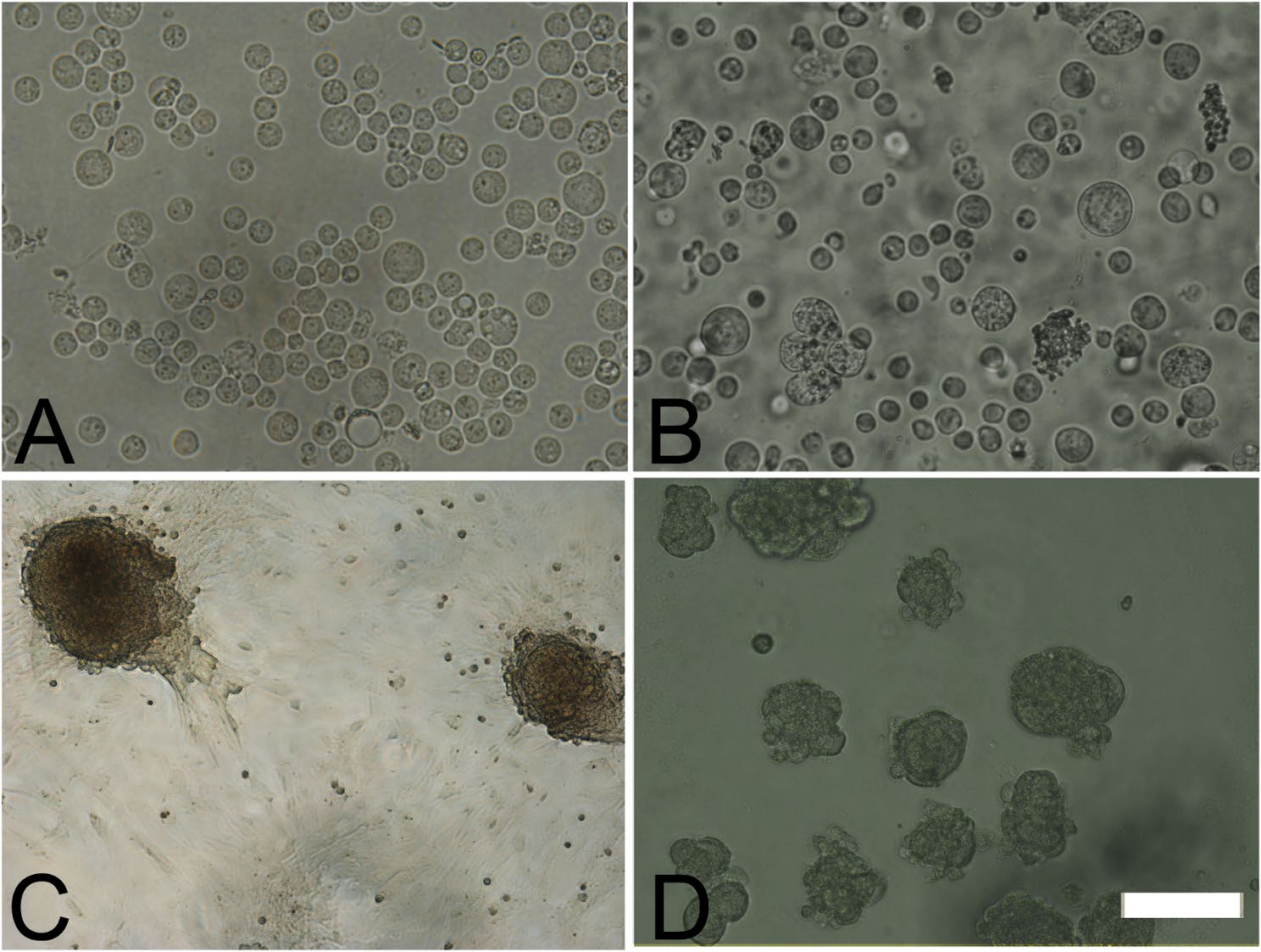
Formation of SSC colonies in primary cultures of neonatal (one week old) and adult (3 month old) mice testicular cells. Cell suspension prepared from the seminiferous tubules of neonatal (**A**) and adult (**B**) mice were followed up for 4 weeks. SSC colonies were formed on day 5 in cultures of germ cells from neonatal (**C**) and adult (**D**). Bar = 20 μm

Primary culture was established from germ cells from neonatal (one week old) and adult (three months old) mouse testes. Cell suspension prepared from the seminiferous tubules of neonatal mouse was plated on gelatin coated plates on the first day. The floating germ cells were replated on day 2 (Fig. 2, A). The testicular somatic cells remained attached to the plate and proliferated rapidly forming a monolayer, while the floating germ cells formed clusters and later transformed into small colonies by day 5 (Fig. 2, C). Similarly, cell suspension from the seminiferous tubules of adult mouse (Fig. 2, B) also generated small clusters on day 4 which transformed into compact SSC colonies by day 5 (Fig. 2, D) respectively. The colonies formed in the cultures of SSCs derived from neonatal testes were larger in size when compared to its counterparts from the adult testes. These SSC colonies were manually transferred onto a new 12 well plate and replenished with fresh SF media on every 7th day and were successfully maintained up to two months.

### Evaluation for stemness in SSC colonies from neonatal testis

**Fig. 3:**
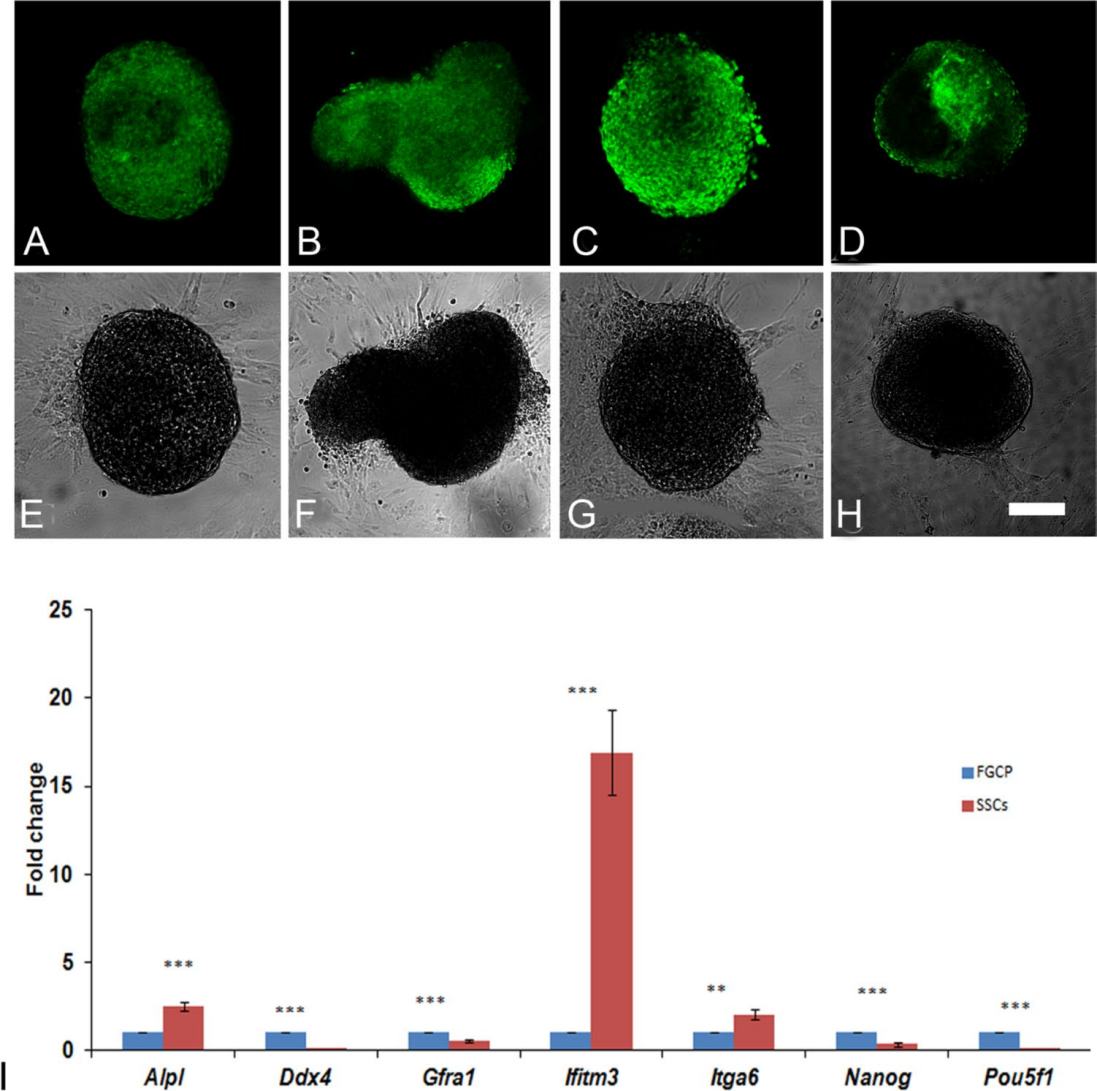
Confocal microscopy of SSC colonies derived from neonatal mice testis in day 24 of culture, showing the expression of GFRA1 (**A**), ITGA6 (**B**) and ALPL (**C**) and CD9 (**D**). The corresponding phase contrast images (**E**-**H**) of the SSC colonies are also shown. Bar = 20 μm. Real time PCR analysis of the expression of stem cell markers and a germ cell marker in SSC colonies on day 24 of culture (**I**). Bar = 20 μm

The stemness of SSC colonies grown in primary culture for 24 days was analyzed by evaluating the expression of a subset of known pluripotency markers using immunofluorescence microscopy. Immunolocalization studies revealed that SSC colonies were positive for GFRA1 (Fig. 3A), ITGA6 (Fig. 3B), ALPL (Fig. 3C) and CD9 (Fig. 3D). The corresponding phase contrast images are shown in Fig. 3, E-H).

The pluripotency/ stemness and germline status of the SSC colonies generated were assessed by evaluating the expression levels of markers such as *Alpl, Gfra1, Itga6, Ifitm3, Nanog, Pou5f1* and *Ddx4* in the floating germ cells and the SSC colonies. The stemness markers *Alpl, Itga6* and *Ifitm3* were upregulated in SSCs colonies, while the key germline marker *Ddx4 and pluripotency markers such as Nanog and Pou5f1* were downregulated significantly (Fig. 3, I). However, the mRNA levels of the SSC marker *Gfra1* was significantly downregulated, which was in contrast with the observation made from our immunocytochemical studies.

**Fig. 4:**
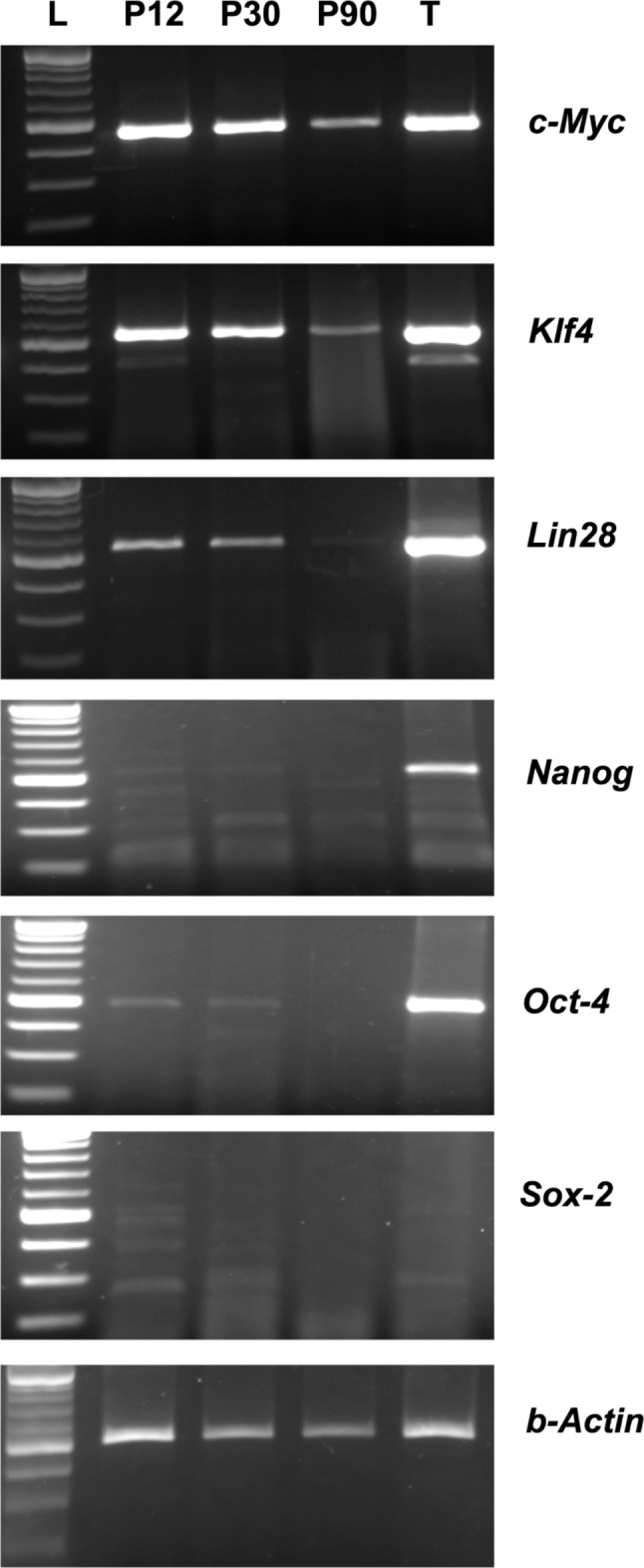
Expression of *c-Myc, Klf4, Nanog, Lin28, Oct4*, and Sox-2 in ES-like cells derived from neonatal (P12), adolescent (P30) and adult (P90) mouse testicular cells after 5–6 days culture in growth factors free DMEM-F12 medium. Postnatal day 8 mouse whole testes (T) used as a positive control. *Actb* was used as an internal control. L-100 bp DNA ladder

The expression levels of key pluripotency markers such as *c-Myc, Klf4, Lin28, Nanog, Oct4* and *Sox2* were analysed in SSC-derived ES-like colonies derived from P12, P30 and P90 mouse testes grown in DMEMF12 medium without any added growth factors. The colonies derived from the SSCs from P12 and P30 testes showed abundance of *c-Myc, Klf4, Lin28*, while *Nanog, Oct4*, and *Sox2* were sparsely expressed. However, ES like colonies derived from SSCs of adult testes showed a reduction in the expression level of *c-Myc, Klf4, Lin28* when compared with the other two groups, whereas *Nanog, Oct4*, and *Sox2* were not detected (Fig. 4).

### SSCs differentiated into embryoid bodies

**Fig. 5:**
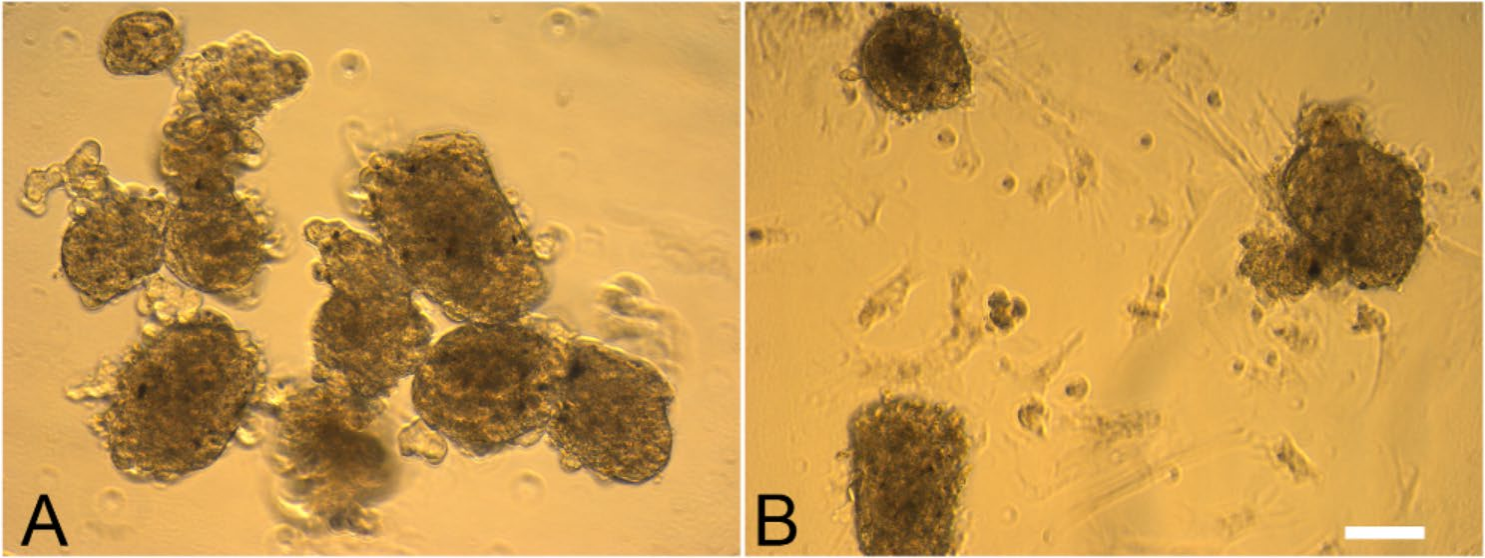
Embryoid body (EB) formation on withdrawal of growth factors. Spermatogonial Stem Cells (SSCs) colonies from neonatal testis (**A**), were cultured for 10 days after removal of growth factors LIF and GDNF (**B**). Bar = 20 μm

**Fig. 6:**
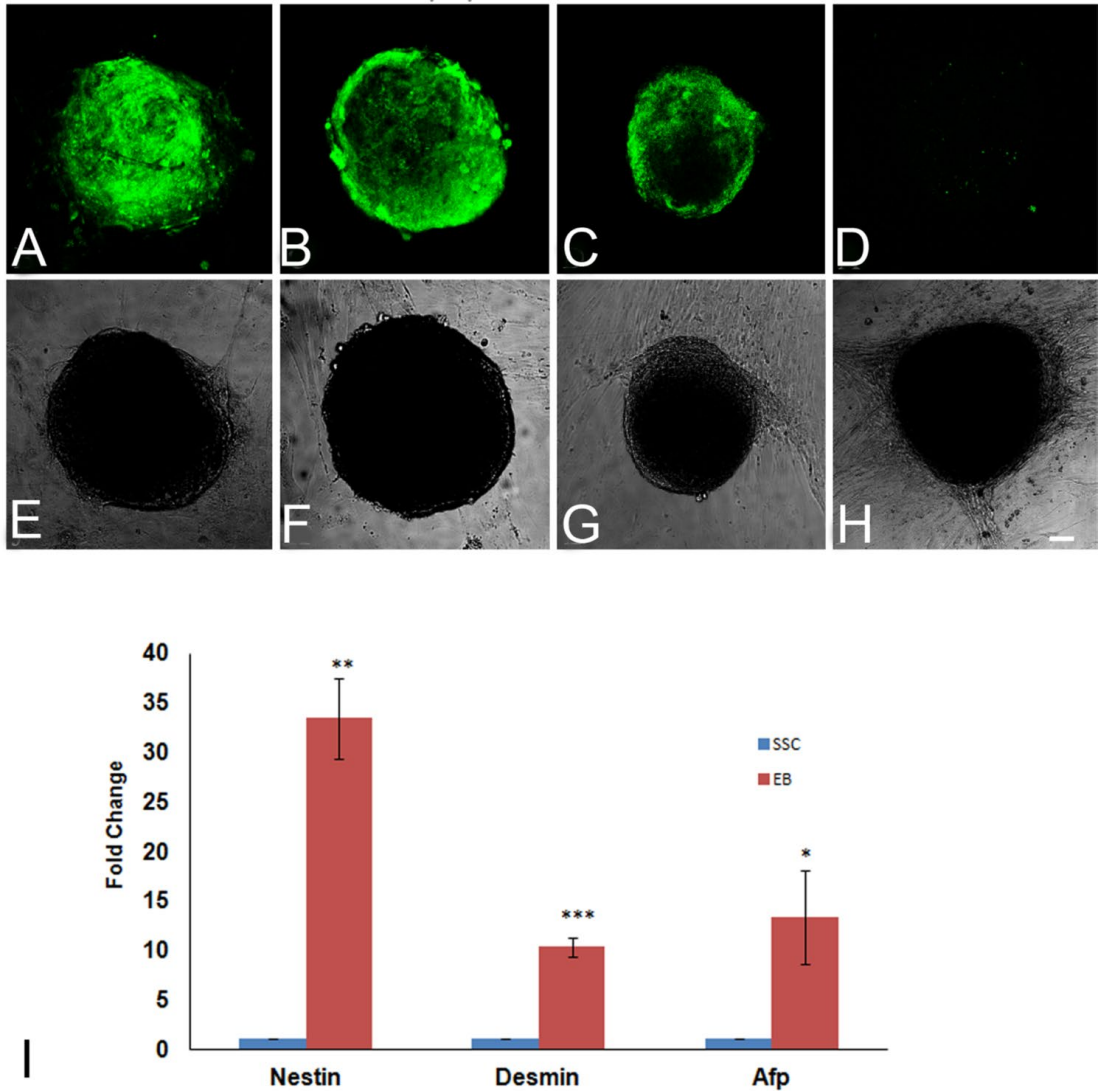
Expression of NESTIN (**A** and **E**), BRACHYURY (**B** and **F**), and PDX-1 (**C** and **G**) embryoid bodies (EBs) derived from neonatal mice testicular cells 10 days after removal of LIF and GDNF, (**D** and **H**) are secondary antibody control. Bar = 20 μm. Real-time PCR analysis of germ layer markers *Nes*, *Des*, *Afp* in SSC-derived EBs 10 days after removal of LIF and GDNF (**I**). *Actb* was used for internal normalization and the expression levels of these genes in EBs were further normalized with their corresponding expression levels in SSCs

Spermatogonial stem cell colonies produced from neonatal mice were maintained in medium containing growth factors LIF and GDNF. The maintenance of these SSC colonies in medium devoid of these growth factors induced differentiation in them. Transformation of SSC colonies (Fig. 5A) into embryoid bodies was observed within 5–7 days after removal of LIF and GDNF in SSCs derived from the neonatal mouse testes (Fig. 5B). The SSC derived embryoid bodies were evaluated for the expression of all the three germ layer markers using immunolocalisation studies. The embryoid bodies expressed NESTIN (Fig. 6A), BRACHYURY (Fig. 6B) and PDX-1 (Fig. 6C) indicating the presence of ectoderm, mesoderm and endoderm layers in them. A negative control, which was probed only with the secondary antibody, is also shown (Fig. 6D). The corresponding DIC images are shown in Fig. 6, E-H. Both the SSC clusters and the embryoid bodies were analyzed by real time PCR for the expression of germ layer markers. The relative quantities of the express of the three germ layer markers (*Nes* for ectoderm, *Des* for mesoderm and *Afp* for endoderm) are shown in Fig. 6, I. We observed 33-fold increase in the expression of *Nes*, 10-fold increase of *Des* and 13-fold increase of *Afp* in the EB-like colonies when compared to SSC colonies (Fig. 6, I).

### SSC colonies and EB-like structures from SSCs from adult testis

**Fig. 7:**
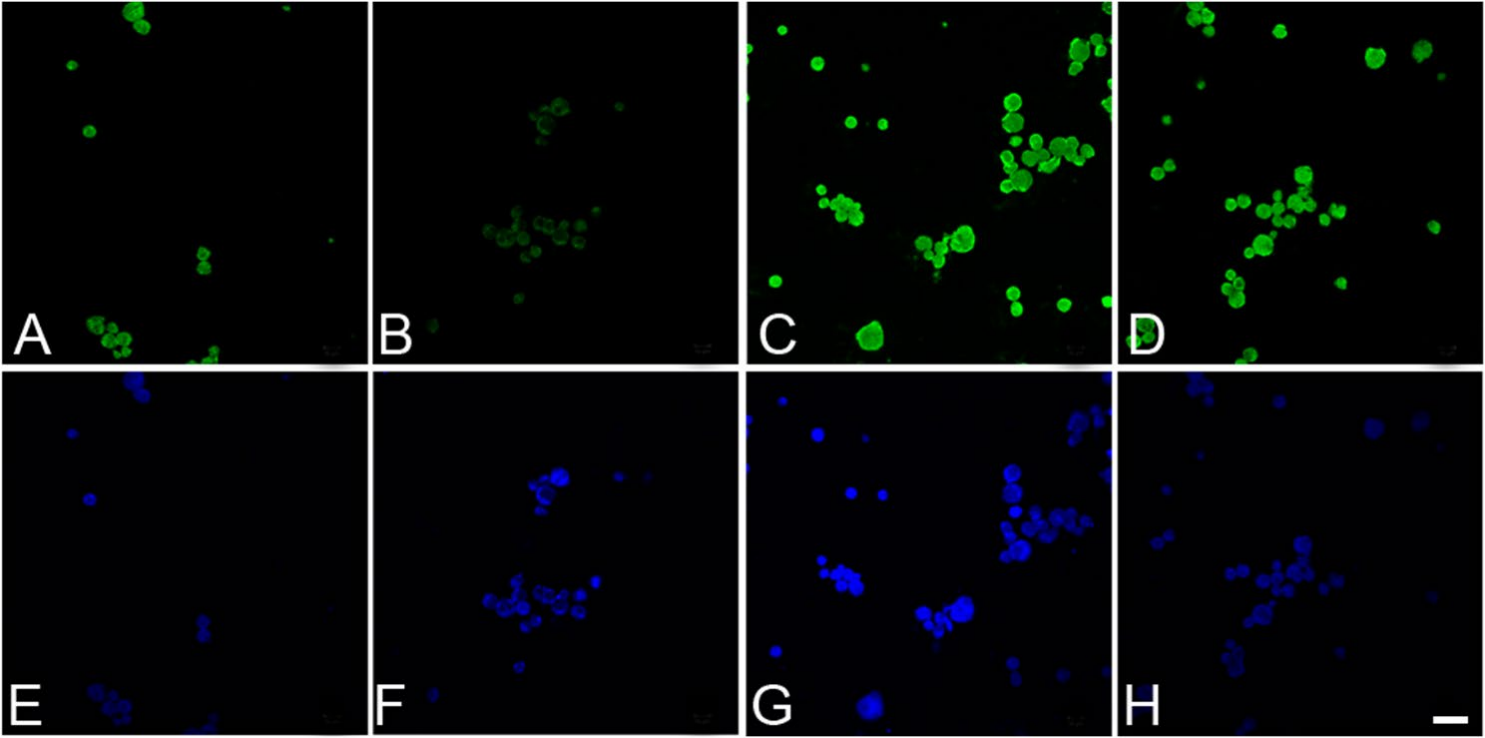
Expression of GFRA1 (**A**), ITGA6 (**B**), ALPL (**C**) and CD9 (**D**) in dispersed cells of SSCs derived from adult mice testicular cells. The corresponding DAPI-stained images are shown in **E**-**H**. Bar = 20 μm

**Fig. 8:**
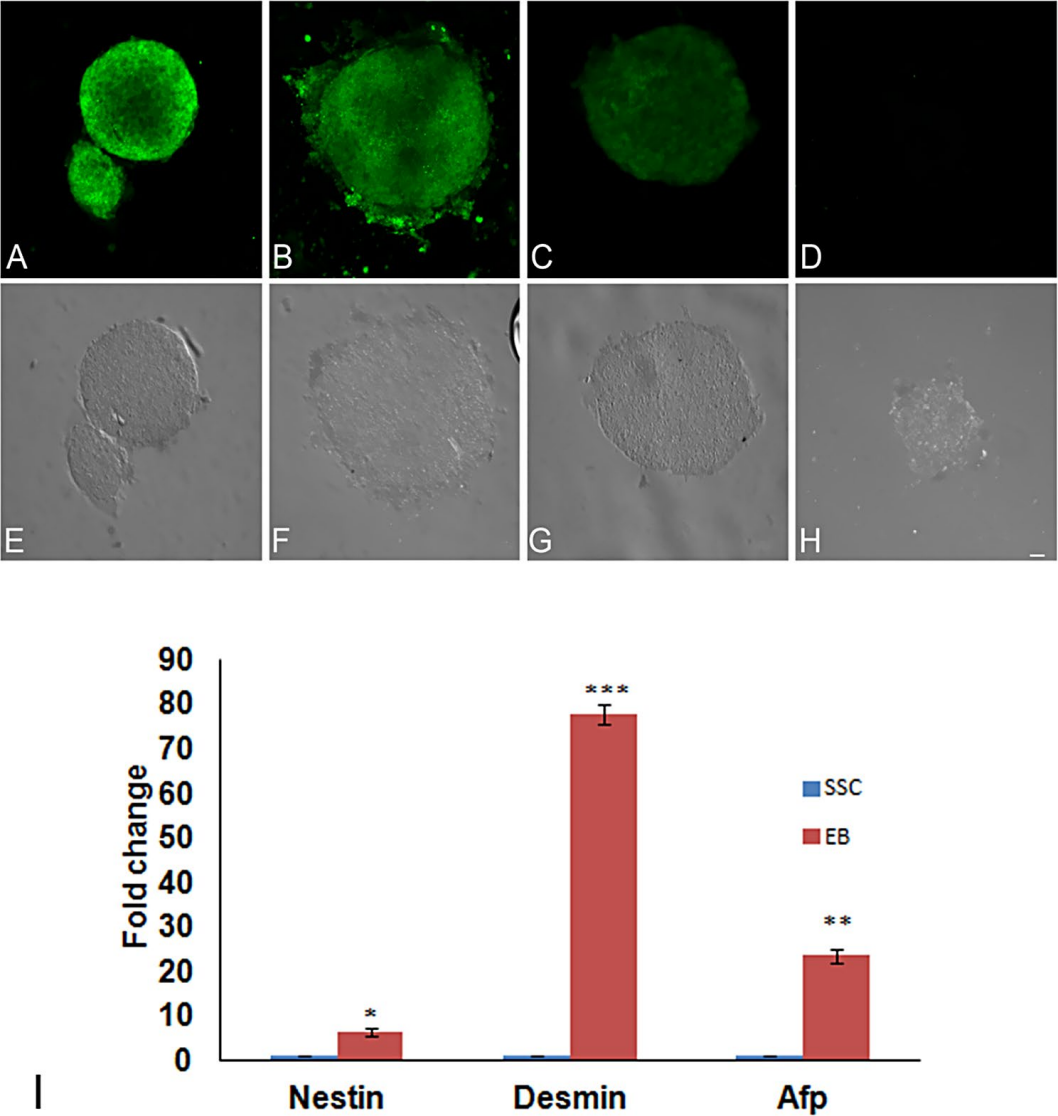
Expression of NESTIN (**A** and **E**), TBXT (BRACHYURY) (**B** and **F**), and PDX-1 (**C** and **G**) embryoid bodies (EBs) derived from adult mice testicular cells 10 days after removal of LIF and GDNF, (**D** and **H**) are secondary antibody control. Bar = 20 μm. Real time PCR analysis of germ layer markers *Nes*, *Des*, *Afp* in SSC-derived EBs 10 days after removal of LIF and GDNF (**I**). *Actb* was used for internal normalization and the expression levels of these genes in EBs were further normalized with their corresponding expression levels in SSCs

SSCs isolated from adult mouse testis produced relatively loose grape-like clusters. Further the dispersed SSCs colony cells stained positive for GFRA1, ITGA6, ALPL and CD9 (Fig. 7, A-D). These cells counter-stained with DAPI are presented in Fig. 7, E-H. The withdrawal of LIF and GDNF from the culture medium induced the formation of compact bodies, which stained positive for NES, BRACHYURY and PDX1 (Fig. 8, A-C). A negative control was also run with the exclusion of primary antibody from the incubation (Fig. 8, D). The corresponding DIC images are shown in Fig. 8, E-H.

Realtime PCR analysis was performed to evaluate the expression levels of the three primary germ layer markers *Nes, Des* and *Afp* in these EB-like structures A significant increase in the relative expression levels of all the three markers in EB-like structures, when compared with that from SSC colonies was observed. Thus, the fold changes in the expression levels of *Nes, Des* and *Afp* in EB-like structures were 4, 86 and 30 respectively (Fig. 8, I).

## Discussion

The long-held notion that spermatogonial stem cells (SSCs) of the testes are unipotent was unravelled by the production of ES-like cells from neonatal mouse testis, which formed teratomas and could also participate in normal embryonic development [21]. Studies on the developmental fate of a single spermatogonial stem cell revealed that conversion of SSC into a pluripotent cell type is accompanied by loss of spermatogenic potential [2]. In line with this observation, this study also demonstrates a significant reduction in the expression of a key germ cell marker *Ddx4* in the SSC-derived ES-like colonies derived from immature testes when cultured without any feeder cells. This result indicates a possible eraser of germ cell imprints in SSCs due to loss of their niche. Hickford et al. had reported that *Ddx4* was crucial for germ cell proliferation and differentiation and its expression was highly regulated by epigenetic modification specifically dimethylation of arginine motifs and CpG islands at its promoter region [35].In addition, studies had reported the upregulation of *Ddx4* expression in germ cells once they enter the gonads, whereas it’s expression was not detected in pluripotent ES, EG or inner cell mass (ICM) cells [36]. However, germ cell colonies could be initiated from ICR or C57BL/6 × DBA/2 F1 (BDF1) mouse strains but not from C57BL/6 or 129/Sv genetic backgrounds. Hence, the derivation of germ cell colonies in vitro depended upon the niche and the genetic background of the mouse strain under study [20]. Later, Guan et al. [22] showed that SSCs from adult mice (C57BL/6, FVB and 129/Ola) testis responded to culture conditions and acquired embryonic stem cell like properties and produced multipotent adult germline stem cells (maGSCs) which could differentiate into three germ layers in vitro. Further, it produced teratomas in immunodeficient mice and could participate in development when injected into blastocysts [22]. Our study demonstrates for the first time the successful generation of ES-like cells from the neonatal and adult testes SSCs from a common mouse strain like Swiss Albino. Also, these SSC-derived ES-like cells could produce EBs comprising of the three primordial germ layers, thereby establishing their pluripotent like state in vitro once again.

The formation of ES-like colonies in germ cell cultures from human testis has been reported [37, 38]. However, the time required for stem cell colony formation was lesser in the germ cell cultures of murine testis compared to those reported for human testicular germ cell cultures reported in these studies. Compact SSC colonies were formed in both neonatal and adult mice testicular cell culture maintained with growth factors (Fig. 2). These SSC colonies remained in pluripotent like state for months when grown in growth factor supplemented medium. Though ES-like clusters formed in SSC cultures from neonatal and adult mouse testes when grown in feeder free and growth factor free media, the efficiency was less and the colonies were unable to maintain their pluripotent nature for longer periods. Plethora of earlier studies had well documented the importance of growth factors in SSC maintenance [39, 40]. These SSC derived ES-like colonies expressed ALPL, GFRA1, ITGA6 and CD9 indicating their stemness [41, 42].

The expression of stemness markers such as *Alpl*, *Itga6* and *Ifitm3* in the SSC derived ES like colonies (Fig. 3, I) indicates their pluripotent status. Moreover, the ES like clusters formed in SSC cultures in growth factor or feeder free media were also positive for c*-Myc, Klf4, Lin24, Oct-4*, *Nanog* and *Sox2*, indicating their inherent pluripotency state. However, the expression of these markers was reduced in ES-like colonies derived from SSCs from adult testes when compared with those from immature (Fig. 4), though it did not affect their differentiation potential. Reduced expression of these pluripotency factors in SSC colonies derived from adult testes might be due to their exposure to the differentiation priming cues produced by the testicular niche and the differentiation primed epigenetic modifications. Recently, Sojoudi et al. reported that mouse SSCs exhibited heterogeneity in colonies formed during in vitro cultures, as they differed in their appearances and molecular marker expression. However, the reason behind this heterogeneity is not understood. CTCF, SMAD3 and SOX2 were important transcription factors predicted to transform the SSCs committed to spermatogenesis into pluripotent form in p53^−/−^ mice in long-term cultures in the absence of exogenous growth factors. SMAD3 is a prerequisite for reprogramming of the SSCs into pluripotent state in long term cultures. Since it was found that p53 knockout affected SMAD3 induction in SSCs, there is a connection between p53 gene expression and pluripotency associated factors. Thus, this study provides a new insight into SSC reprogramming mechanism and tumorogenesis of GSCs [43]. The expression of pluripotency markers SSEA-4, OCT-4 and SOX 2 was detected in human spermatogonial stem cell derived ES-like cells [39].

The differentiation potentials of the stem cell colonies derived from the mouse testis were studied by withdrawing growth factors LIF and GDNF from the culture medium. Differentiation could be induced in ES cells following LIF withdrawal leading to the formation of embryoid bodies [40]. The withdrawal of LIF and GDNF from the culture medium was based on reports showing that the addition of LIF to SSC cultures is superfluous and that GDNF is required for SSC maintenance [41]. GDNF produced by Sertoli cells and endothelial cells in the testis regulates the expression of genes implicated in the maintenance of self-renewing state and/or prevention of differentiation of SSCs (A_undiff_) which include *Nanos2, Etv5, Lhx1, T(Brachyury), Bcl6b, Id4* and *Cxcr4* [44–49] The loss or withdrawal of GDNF might downregulate the expression of self-renewal genes under its signalling or regulation. Therefore, the down regulation of self-renewal associated genes in SSCs cultured in growth factor deprived condition appears to lead to their differentiation. In testis, SSC differentiation is mediated by retinoic acid (RA) signalling and canonical WNT signalling. In the absence of such differentiation factors in the culture medium, the formation of EB like bodies from SSC colonies was rather spontaneous and their differentiation induction appears to be primarily due to the loss of in vitro niche required for spermatogenic differentiation. Further, such EB like bodies developed from SSC colonies derived from neonatal and adult mouse testes upon removal of growth factors expressed ectodermal (*Nes*), mesodermal (BRACHYURY (TBXT) and *Des*) and endodermal (*Afp* and PDX-1) cell linage markers (Figs. 5 and 7) suggesting natural transdifferentiation of SSC colonies considering the upregulation of *Ifitm3* and downregulation of *Pou5f1, Nanog* and *Gfra1* in cultured SSC colonies (Fig. 3I). The use of NES [42], TBXT [50], DES [43], AFP [50] and PDX-1 [44] as germ layer markers has been reported. Trans-differentiation is a form of lineage reprogramming or cellular process which involves the direct conversion of one type of differentiated cell into another distinct cell type from its original lineage without going through an intermediate pluripotent state, reflecting a high level of cellular plasticity [51, 52]. This transformation involves the direct conversion of one specialized cell type into another, often from one tissue or germ layer to another through specific molecular changes by alterations in gene expression, epigenetic modifications and signalling pathway activation that drives the cells toward the new fate [53–55].

During natural transdifferentiation, first the cell dedifferentiates and later, the innate developmental programme is activated allowing the cell to differentiate into a new lineage [56]. Newt lens regeneration perfectly illustrates the naturally occurring transdifferentiation process during which, pigmented epithelial cells (PECs) dedifferentiates, proliferates by re-entering into cell cycle and finally differentiates into crystallin expressing mature lens cell [57, 58]. In the present study, *Ifitm3* which is expressed in migrating primordial germ cells (PGCs) and is implicated to have roles in germ cell development [59] was found to be upregulated during in vitro SSC culture, suggesting possible dedifferentiation of SSC to an intermediate primitive state and those cells which in turn transdifferentiates into all three germ lineages. Further, involvement of BMP and upregulation of Wnt signalling during transdifferentiation from cornea to lens in Xenopus laevis was reported [60]. BMPs and WNT mediated signalling are implicated in early and late germ cell development like PGC induction, proliferation, migration and gametogenesis [61, 62]. Therefore, the embryoid body like body formation from SSC colonies might also have utilized BMP and WNT mediated dedifferentiation and transdifferentiation processes. A comparative analysis of small RNA signatures from SSCs, Sertoli cells, developing germ cells, ESCs and MSCs using high throughput sequencing revealed that the miRNA signature in mouse SSCs were similar to those of ESCs [45]. Thus, we could establish ES-like clusters from germ cells isolated from the testis of neonatal and adult Swiss albino mice which expressed pluripotency genes and upon induction of differentiation formed embryoid bodies similar to those derived from ES cells.

## Conclusion

GSCs colonized in primary germ cell culture from Swiss albino mice testicular cells and its stemness was confirmed by demonstrating the expression of pluripotency markers. Majority of the studies on GSCs till date employed GS cells established from transgenic mouse lines. This study reports for the first-time isolation of GSCs from Swiss albino mice testicular cells which could form colonies, maintain stemness and form EBs upon induction of differentiation. Further, both neonatal and adult mouse-derived GSCs have comparable ability to make SSC colonies and EB-like bodies and have similar gene expression. The germline stem cell derived embryoid bodies were comparable to those formed by embryonic stem cells in vitro. The authenticity and differentiation potential of the germline stem cell derived embryoid bodies was confirmed by localizing germ layer markers to these embryoid bodies. Transplantation assays and lineage specific differentiation of these testis derived stem cells are underway.

### Electronic supplementary material

Supplementary Material 1

## Data Availability

Not applicable.

## Declarations

### Author information

PGK was a senior scientist heading the Molecular Reproduction Lab-1 at Rajiv Gandhi Centre for Biotechnology until his superannuation on 31 July 2022 and is currently an Emeritus Scientist of Indian Council of Medical Research, New Delhi at Department of Biotechnology, University of Kerala, Thiruvananthapuram. He is a Life member of professional societies including Indian Society for the Study of Reproduction and Fertility (ISSRF), Society of Biological Chemists India (SBCI), Indian Society of Cell Biology (ISCB) and Society for Reproductive Biology and Comparative Endocrinology (SRBCE). SI is a post-doctoral fellow; AND was a PhD student; MS is a Senior Research Fellow; JS and SRK are doctoral students and AB was a short-term research intern.

## Abbreviations

ALP: Alkaline Phosphatase
BSA: bovine serum albumin
CNV: copy number variation
DEPC: Diethyl pyrocarbonate
DMEM: Dulbecco’s Modified Eagle’s Medium
DNA: Deoxy ribonucleic acid
EB: Embryoid body
ES: Embryonic Stem Cell
DAPI: 4’,6-diamidino-2-phenylindole
FBS: fetal bovine serum;FGF:fibroblast growth factor
FGCP: floating germ cell population
GDNF: glial derived neurotrophic factor
GSC: germline stem cell
iPSC: induced Pluripotent Stem cell
LIF: leukemia inhibiting factor
MEM: minimum essential medium
M-MuLV: Moloney Murine Leukemia Virus
NEAA: non-essential amino acid
NTP: nucleotide triphosphate
OCT: Octamer binding protein
PBS: Phosphate buffered saline
PCR: polymerase chain reaction
PDX: Pancreatic and duodenal homeobox 1
PGC: primordial germ cell
PI: propidium iodide
RNA: ribonucleic acid
RT: room temperature
SSC: Spermatogonial Stem Cell

## References

[1] Mousaei Ghasroldasht M, Seok J, Park HS, Liakath Ali FB, Al-Hendy A. Stem cell therapy: from idea to clinical practice. Int J Mol Sci. 2022;23(5). 10.3390/ijms23052850 PMC8911494 35269990

[2] Kanatsu-Shinohara M, Lee J, Inoue K, Ogonuki N, Miki H, Toyokuni S (2008). Pluripotency of a single spermatogonial stem cell in mice. Biol Reprod.

[3] Durnaoglu S, Genc S, Genc K (2011). Patient-specific pluripotent stem cells in neurological diseases. Stem Cells Int.

[4] Takahashi K, Yamanaka S (2006). Induction of pluripotent stem cells from mouse embryonic and adult fibroblast cultures by defined factors. Cell.

[5] Draper JS, Smith K, Gokhale P, Moore HD, Maltby E, Johnson J (2004). Recurrent gain of chromosomes 17q and 12 in cultured human embryonic stem cells. Nat Biotechnol.

[6] Baker DE, Harrison NJ, Maltby E, Smith K, Moore HD, Shaw PJ (2007). Adaptation to culture of human embryonic stem cells and oncogenesis in vivo. Nat Biotechnol.

[7] Mayshar Y, Ben-David U, Lavon N, Biancotti JC, Yakir B, Clark AT (2010). Identification and classification of chromosomal aberrations in human induced pluripotent stem cells. Cell Stem Cell.

[8] Laurent LC, Ulitsky I, Slavin I, Tran H, Schork A, Morey R (2011). Dynamic changes in the copy number of pluripotency and cell proliferation genes in human ESCs and iPSCs during reprogramming and time in culture. Cell Stem Cell.

[9] Hussein SM, Batada NN, Vuoristo S, Ching RW, Autio R, Närvä E (2011). Copy number variation and selection during reprogramming to pluripotency. Nature.

[10] Miura K, Okada Y, Aoi T, Okada A, Takahashi K, Okita K (2009). Variation in the safety of induced pluripotent stem cell lines. Nat Biotechnol.

[11] Gutierrez-Aranda I, Ramos-Mejia V, Bueno C, Munoz-Lopez M, Real PJ, Mácia A (2010). Human induced pluripotent stem cells develop teratoma more efficiently and faster than human embryonic stem cells regardless the site of injection. Stem Cells.

[12] Bar-Nur O, Russ HA, Efrat S, Benvenisty N (2011). Epigenetic memory and preferential lineage-specific differentiation in induced pluripotent stem cells derived from human pancreatic islet beta cells. Cell Stem Cell.

[13] Dahlke J, Schott JW, Vollmer Barbosa P, Klatt D, Selich A, Lachmann N et al. Efficient genetic safety switches for future application of iPSC-Derived cell transplants. J Personalized Med. 2021;11(6). 10.3390/jpm11060565 PMC8234706 34204193

[14] Seydoux G, Braun RE (2006). Pathway to totipotency: lessons from germ cells. Cell.

[15] Kurimoto K, Yamaji M, Seki Y, Saitou M (2008). Specification of the germ cell lineage in mice: a process orchestrated by the PR-domain proteins, Blimp1 and Prdm14. Cell Cycle (Georgetown Tex).

[16] Yao C, Yao R, Luo H, Shuai L (2022). Germline specification from pluripotent stem cells. Stem Cell Res Ther.

[17] Tegelenbosch RA, de Rooij DG (1993). A quantitative study of spermatogonial multiplication and stem cell renewal in the C3H/101 F1 hybrid mouse. Mutat Res.

[18] Puri MC, Nagy A (2012). Concise review: embryonic stem cells versus induced pluripotent stem cells: the game is on. Stem Cells.

[19] McLaren A (2001). Ethical and social considerations of stem cell research. Nature.

[20] Kanatsu-Shinohara M, Ogonuki N, Inoue K, Miki H, Ogura A, Toyokuni S (2003). Long-term proliferation in culture and germline transmission of mouse male germline stem cells. Biol Reprod.

[21] Kanatsu-Shinohara M, Inoue K, Lee J, Yoshimoto M, Ogonuki N, Miki H (2004). Generation of pluripotent stem cells from neonatal mouse testis. Cell.

[22] Guan K, Nayernia K, Maier LS, Wagner S, Dressel R, Lee JH (2006). Pluripotency of spermatogonial stem cells from adult mouse testis. Nature.

[23] Golestaneh N, Kokkinaki M, Pant D, Jiang J, DeStefano D, Fernandez-Bueno C (2009). Pluripotent stem cells derived from adult human testes. Stem Cells Dev.

[24] Zvetkova I, Apedaile A, Ramsahoye B, Mermoud JE, Crompton LA, John R (2005). Global hypomethylation of the genome in XX embryonic stem cells. Nat Genet.

[25] Li Y, Wang X, Feng X, Liao S, Zhang D, Cui X (2014). Generation of male germ cells from mouse induced pluripotent stem cells in vitro. Stem cell Res.

[26] Falciatori I, Lillard-Wetherell K, Wu Z, Hamra FK, Garbers DL (2008). Deriving mouse spermatogonial stem cell lines. (Clifton NJ).

[27] Gassei K, Ehmcke J, Schlatt S (2009). Efficient enrichment of undifferentiated GFR alpha 1 + spermatogonia from immature rat testis by magnetic activated cell sorting. Cell Tissue Res.

[28] Kanatsu-Shinohara M, Shinohara T (2010). Germline modification using mouse spermatogonial stem cells. Methods Enzymol.

[29] Kurosawa H (2007). Methods for inducing embryoid body formation: in vitro differentiation system of embryonic stem cells. J Biosci Bioeng.

[30] Thomas C, Doetschman HE (1985). Margot Katz, Werner Schmidt and Rolf Kemler. The in vitro development of blastocyst-derived embryonic stem cell lines: formation of visceral yolk sac, blood islands and myocardium. J Experimental Morphology.

[31] Desbaillets I, Ziegler U, Groscurth P, Gassmann M (2004). Embryoid bodies: an in Vitro Model of Mouse Embryogenesis. Exp Physiol.

[32] Liu X, Huang J, Chen T, Wang Y, Xin S, Li J (2008). Yamanaka factors critically regulate the developmental signaling network in mouse embryonic stem cells. Cell Res.

[33] Yu J, Vodyanik MA, Smuga-Otto K, Antosiewicz-Bourget J, Frane JL, Tian S (2007). Induced pluripotent stem cell lines derived from human somatic cells. Science.

[34] Thomson M, Liu SJ, Zou LN, Smith Z, Meissner A, Ramanathan S (2011). Pluripotency factors in embryonic stem cells regulate differentiation into germ layers. Cell.

[35] Hickford DE, Frankenberg S, Pask AJ, Shaw G, Renfree MB (2011). DDX4 (VASA) is conserved in germ cell development in marsupials and monotremes. Biol Reprod.

[36] Bowles J, Koopman P (2010). Sex determination in mammalian germ cells: extrinsic versus intrinsic factors. Reproduction.

[37] Mizrak SC, Chikhovskaya JV, Sadri-Ardekani H, van Daalen S, Korver CM, Hovingh SE (2010). Embryonic stem cell-like cells derived from adult human testis. Hum Reprod (Oxford England).

[38] Sadri-Ardekani H, Mizrak SC, van Daalen SK, Korver CM, Roepers-Gajadien HL, Koruji M (2009). Propagation of human spermatogonial stem cells in vitro. JAMA.

[39] Jeong D, McLean DJ, Griswold MD (2003). Long-term culture and transplantation of murine testicular germ cells. J Androl.

[40] Wang P, Suo LJ, Wang YF, Shang H, Li GX, Hu JH (2014). Effects of GDNF and LIF on mouse spermatogonial stem cells proliferation in vitro. Cytotechnology.

[41] Kanatsu-Shinohara M, Toyokuni S, Shinohara T (2004). CD9 is a surface marker on mouse and rat male germline stem cells. Biol Reprod.

[42] Oka M, Tagoku K, Russell TL, Nakano Y, Hamazaki T, Meyer EM (2002). CD9 is associated with leukemia inhibitory factor-mediated maintenance of embryonic stem cells. Mol Biol Cell.

[43] Liu S, Wei R, Liu H, Liu R, Li P, Zhang X (2022). Analysis of chromatin accessibility in p53 deficient spermatogonial stem cells for high frequency transformation into pluripotent state. Cell Prolif.

[44] Chen C, Ouyang W, Grigura V, Zhou Q, Carnes K, Lim H (2005). ERM is required for transcriptional control of the spermatogonial stem cell niche. Nature.

[45] Oatley JM, Avarbock MR, Telaranta AI, Fearon DT, Brinster RL (2006). Identifying genes important for spermatogonial stem cell self-renewal and survival. Proc Natl Acad Sci USA.

[46] Oatley JM, Avarbock MR, Brinster RL (2007). Glial cell line-derived neurotrophic factor regulation of genes essential for self-renewal of mouse spermatogonial stem cells is dependent on src family kinase signaling. J Biol Chem.

[47] Oatley MJ, Kaucher AV, Racicot KE, Oatley JM (2011). Inhibitor of DNA binding 4 is expressed selectively by single spermatogonia in the male germline and regulates the self-renewal of spermatogonial stem cells in mice. Biol Reprod.

[48] Sada A, Hasegawa K, Pin PH, Saga Y (2012). NANOS2 acts downstream of glial cell line-derived neurotrophic factor signaling to suppress differentiation of spermatogonial stem cells. Stem Cells.

[49] Yang QE, Kim D, Kaucher A, Oatley MJ, Oatley JM (2013). CXCL12-CXCR4 signaling is required for the maintenance of mouse spermatogonial stem cells. J Cell Sci.

[50] Sojoudi K, Azizi H, Skutella T (2023). A Fundamental Research in In Vitro Spermatogonial Stem Cell Culturing: what are clump cells?. Cell Reprogram.

[51] Clayton RM (1982). The molecular basis for competence, determination and transdifferentiation: a hypothesis. Adv Exp Med Biol.

[52] Selman K, Kafatos FC (1974). Transdifferentiation in the labial gland of silk moths: is DNA required for cellular metamorphosis?. Cell Differ.

[53] Li WC, Yu WY, Quinlan JM, Burke ZD, Tosh D (2005). The molecular basis of transdifferentiation. J Cell Mol Med.

[54] Eguizabal C, Montserrat N, Veiga A, Izpisua Belmonte JC (2013). Dedifferentiation, transdifferentiation, and reprogramming: future directions in regenerative medicine. Semin Reprod Med.

[55] Cieślar-Pobuda A, Knoflach V, Ringh MV, Stark J, Likus W, Siemianowicz K (2017). Transdifferentiation and reprogramming: overview of the processes, their similarities and differences. Biochim et Biophys acta Mol cell Res.

[56] Jopling C, Boue S, Izpisua Belmonte JC (2011). Dedifferentiation, transdifferentiation and reprogramming: three routes to regeneration. Nat Rev Mol Cell Biol.

[57] Tsonis PA, Madhavan M, Tancous EE, Del Rio-Tsonis K (2004). A newt’s eye view of lens regeneration. Int J Dev Biol.

[58] Maki N, Martinson J, Nishimura O, Tarui H, Meller J, Tsonis PA (2010). Expression profiles during dedifferentiation in newt lens regeneration revealed by expressed sequence tags. Mol Vis.

[59] Tanaka SS, Yamaguchi YL, Tsoi B, Lickert H, Tam PP (2005). IFITM/Mil/fragilis family proteins IFITM1 and IFITM3 play distinct roles in mouse primordial germ cell homing and repulsion. Dev Cell.

[60] Day RC, Beck CW (2011). Transdifferentiation from cornea to lens in Xenopus laevis depends on BMP signalling and involves upregulation of wnt signalling. BMC Dev Biol.

[61] Lochab AK, Extavour CG (2017). Bone morphogenetic protein (BMP) signaling in animal reproductive system development and function. Dev Biol.

[62] Cantú AV, Laird DJ (2017). Primordial germ cell migration and the wnt signaling pathway. Anim Reprod.

