# Supplementary figures and images for: Expression profiling of stemness markers in testicular germline stem cells from neonatal and adult Swiss albino mice during their transdifferentiation in vitro
Source: Stem Cell Res Ther. 2024 Apr 1;15:93. doi: 10.1186/s13287-024-03701-8 (PMC10985951; doi:10.1186/s13287-024-03701-8)

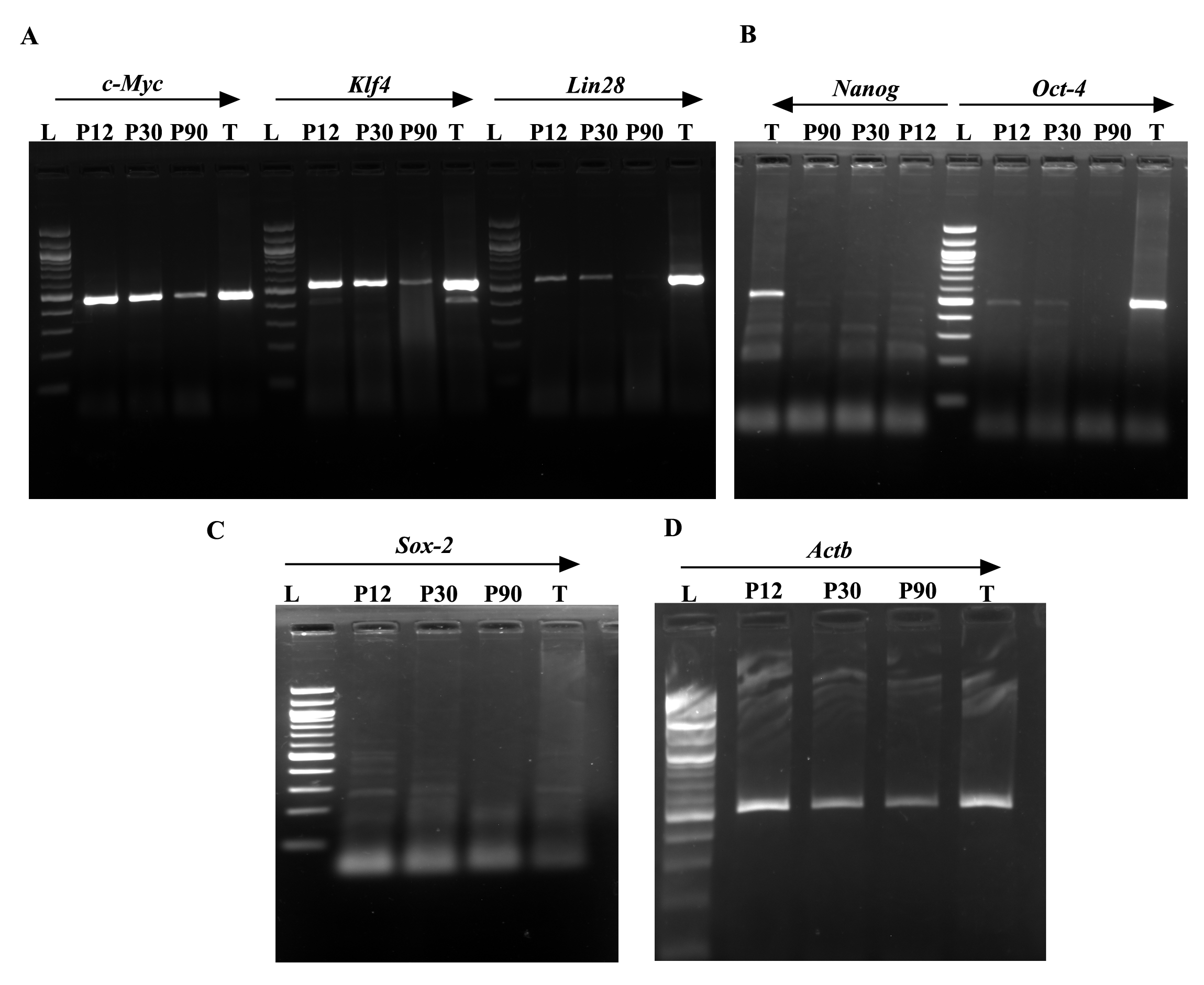

Supplement: Supplementary file 1 — Supplementary Material 1 [file 13287_2024_3701_MOESM1_ESM.tif]
